# Supplementary material for: The Impact of Women’s Agency on Accessing and Using Maternal Healthcare Services: A Systematic Review and Meta-Analysis
Source: Int J Environ Res Public Health. 2023 Feb 23;20(5):3966. doi: 10.3390/ijerph20053966 (PMC10002172; doi:10.3390/ijerph20053966)
Supplement: Supplementary file 1 [file ijerph-20-03966-s001.zip › ijerph-2141587-supplementary.pptx]

## Slide 1
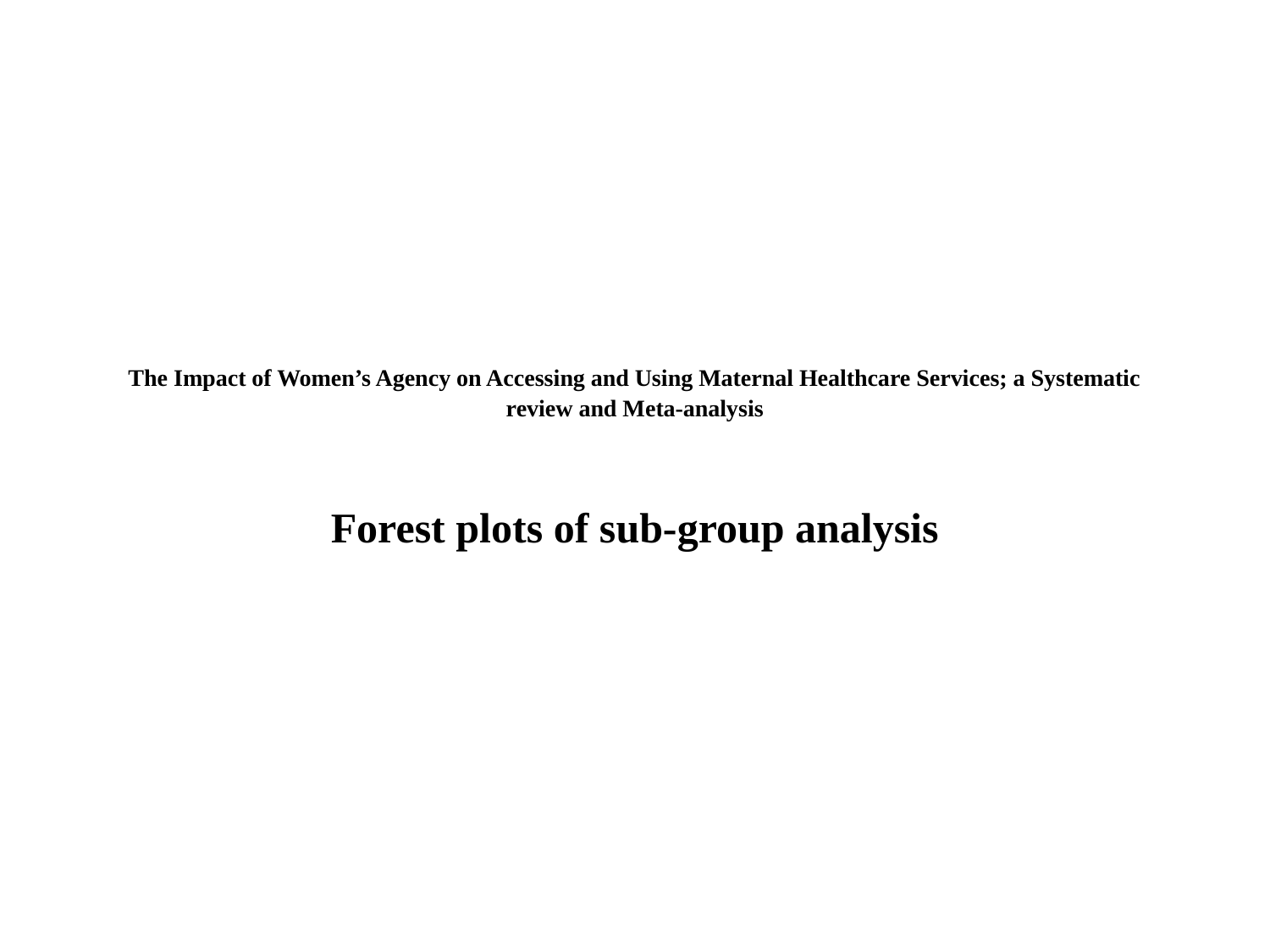

# The Impact of Women’s Agency on Accessing and Using Maternal Healthcare Services; a Systematic review and Meta-analysis
Forest plots of sub-group analysis

## Slide 2
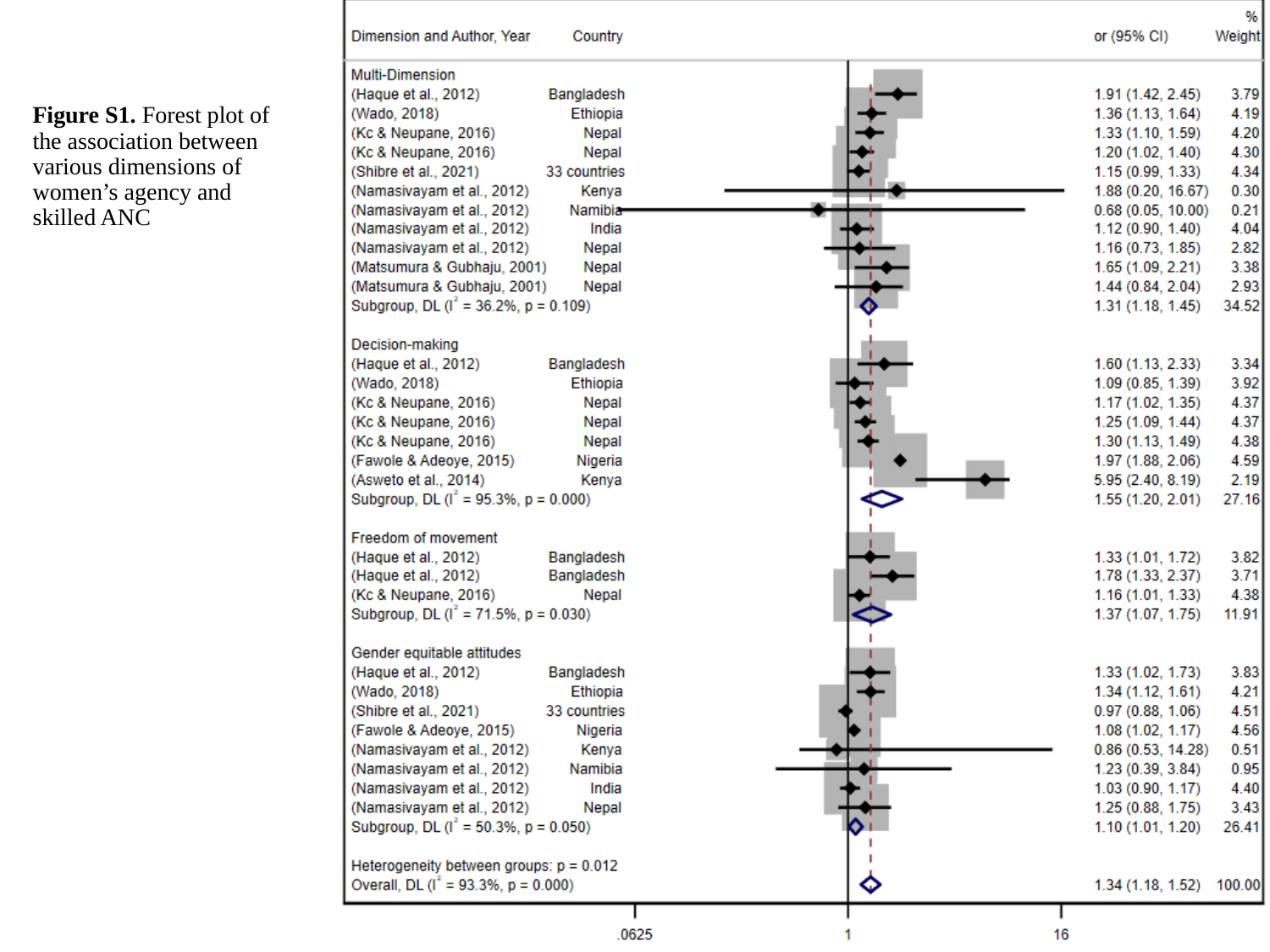

# Figure S1. Forest plot of the association between various dimensions of women’s agency and skilled ANC

## Slide 3
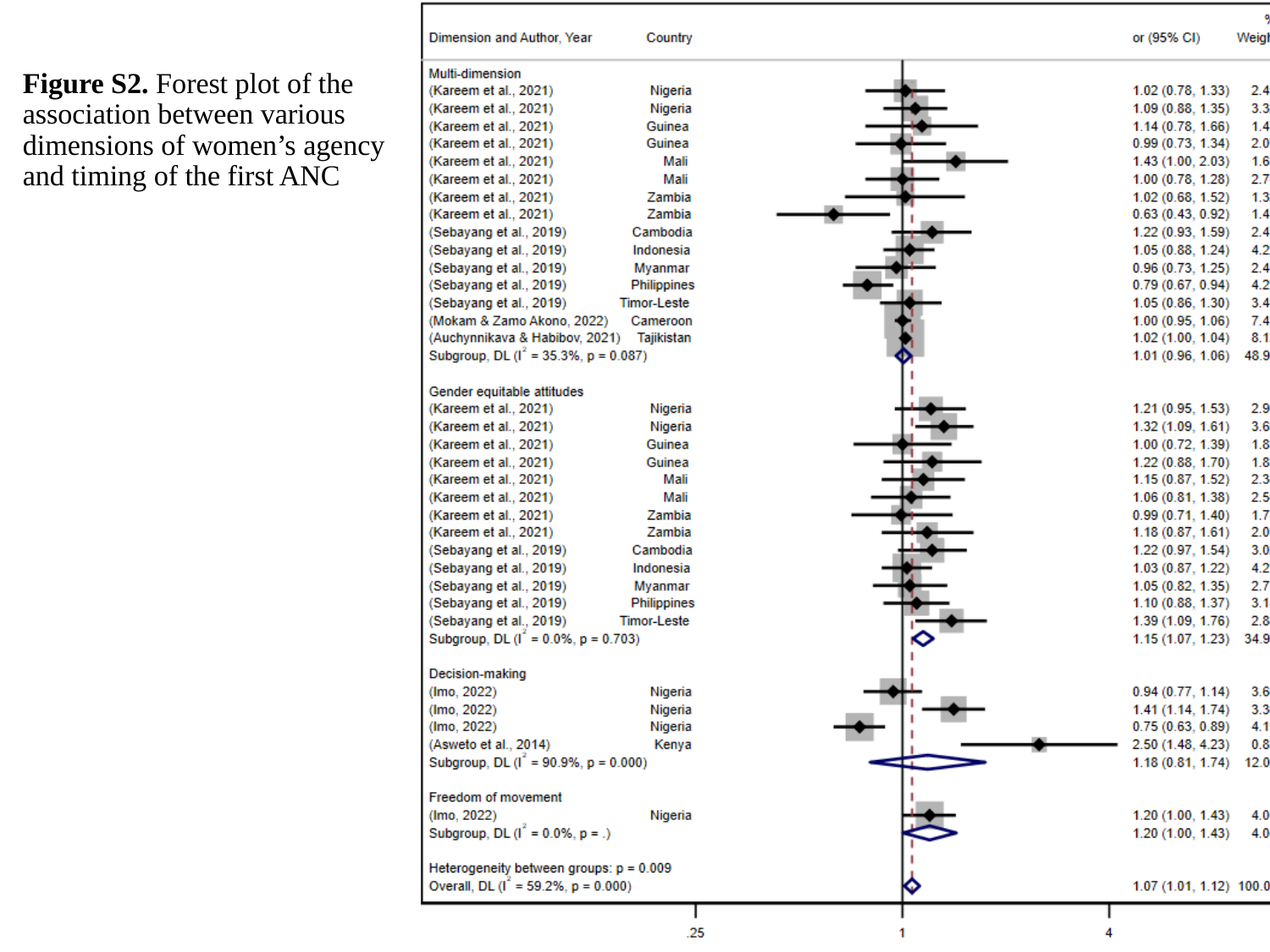

# Figure S2. Forest plot of the association between various dimensions of women’s agency and timing of the first ANC

## Slide 4
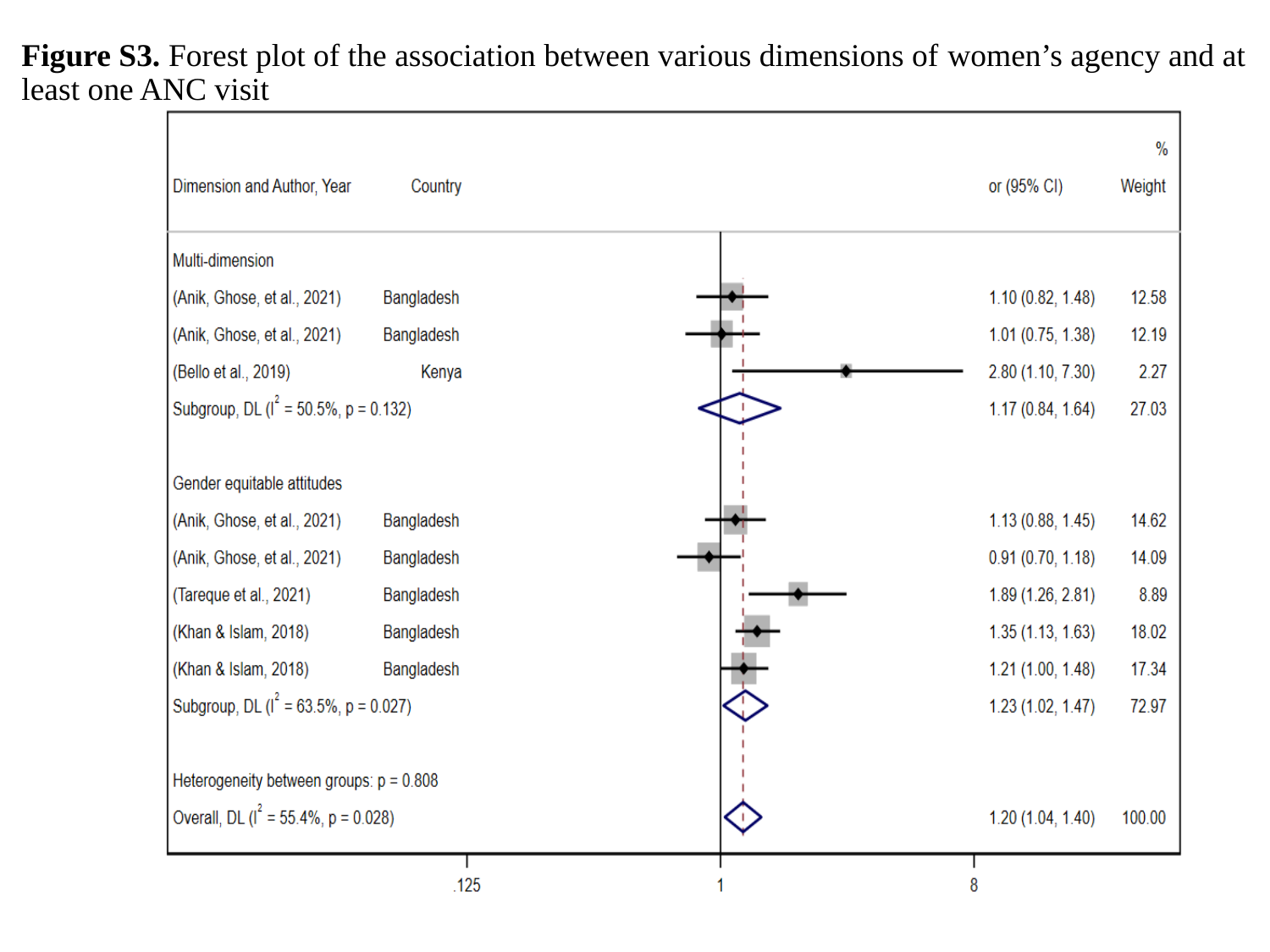

# Figure S3. Forest plot of the association between various dimensions of women’s agency and at least one ANC visit

## Slide 5
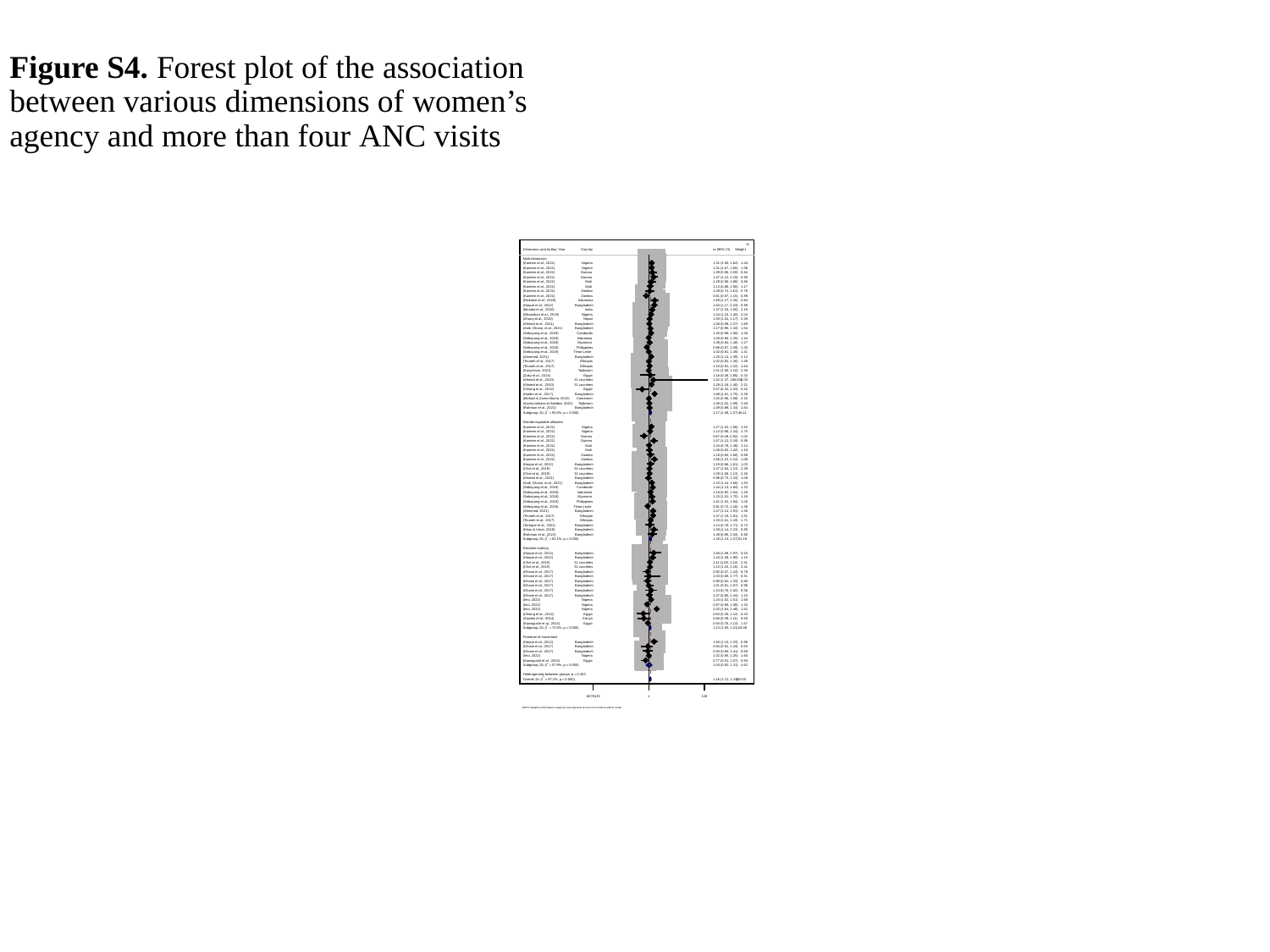

# Figure S4. Forest plot of the association between various dimensions of women’s agency and more than four ANC visits

## Slide 6
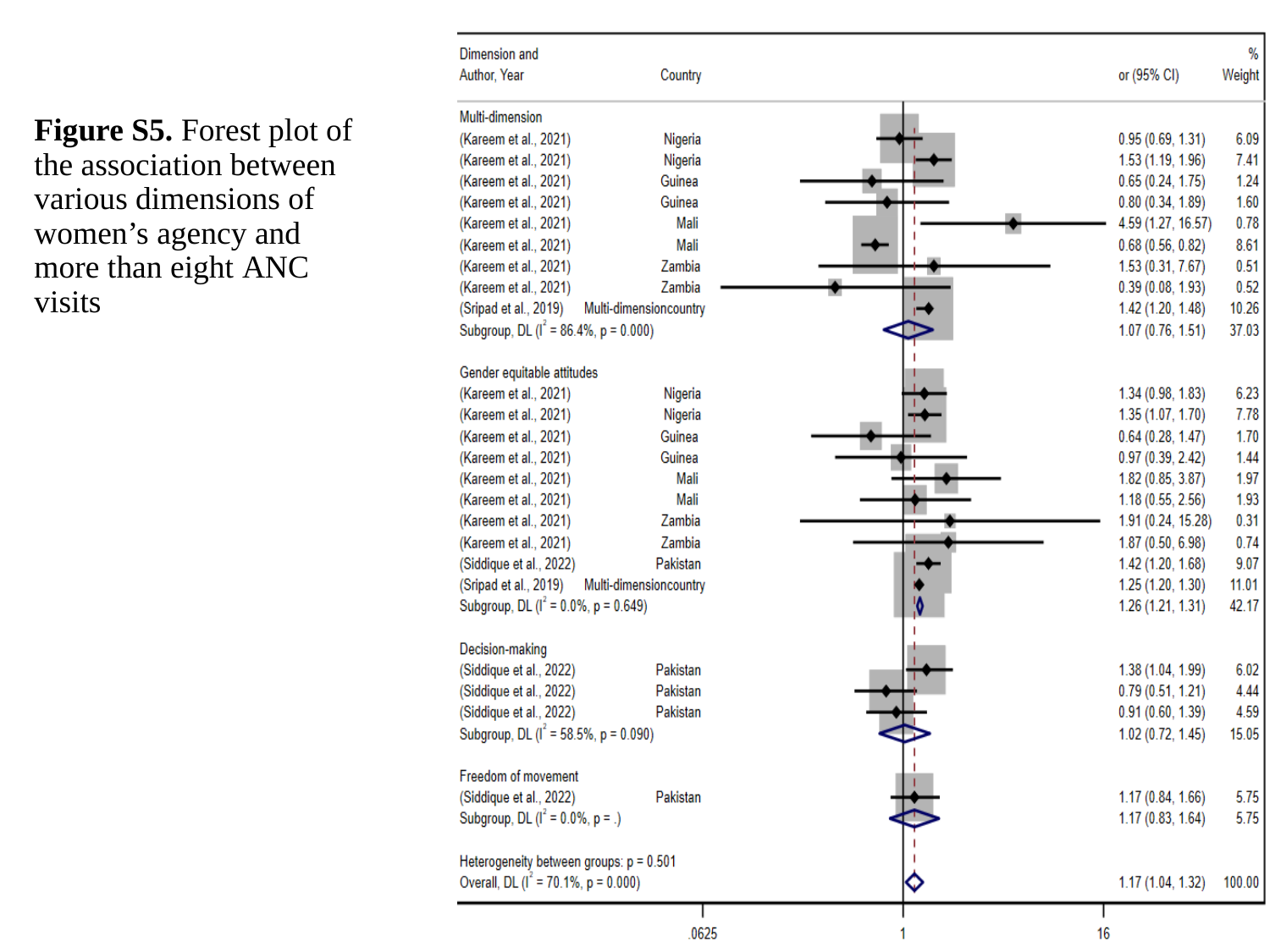

# Figure S5. Forest plot of the association between various dimensions of women’s agency and more than eight ANC visits

## Slide 7
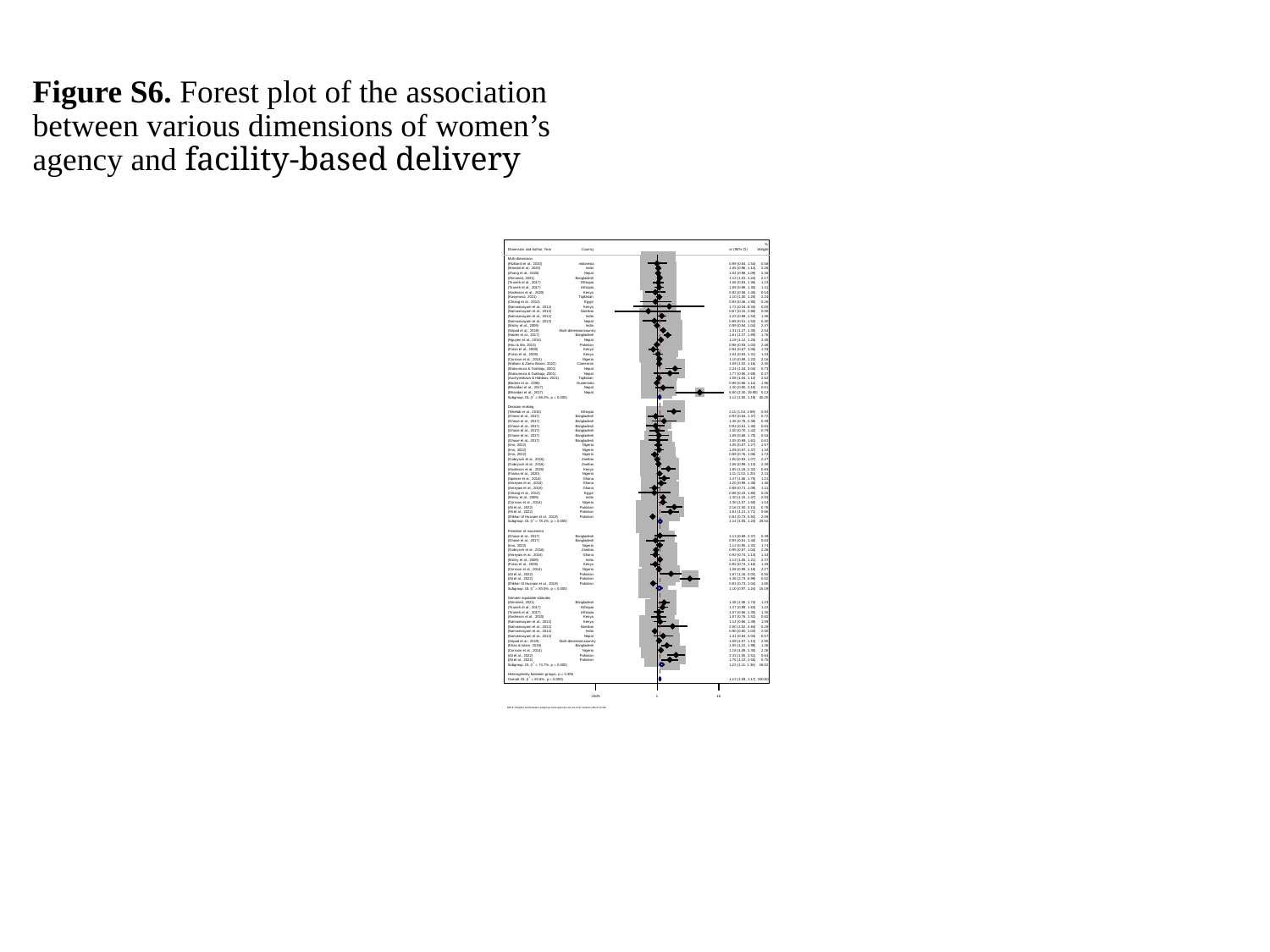

# Figure S6. Forest plot of the association between various dimensions of women’s agency and facility-based delivery

## Slide 8
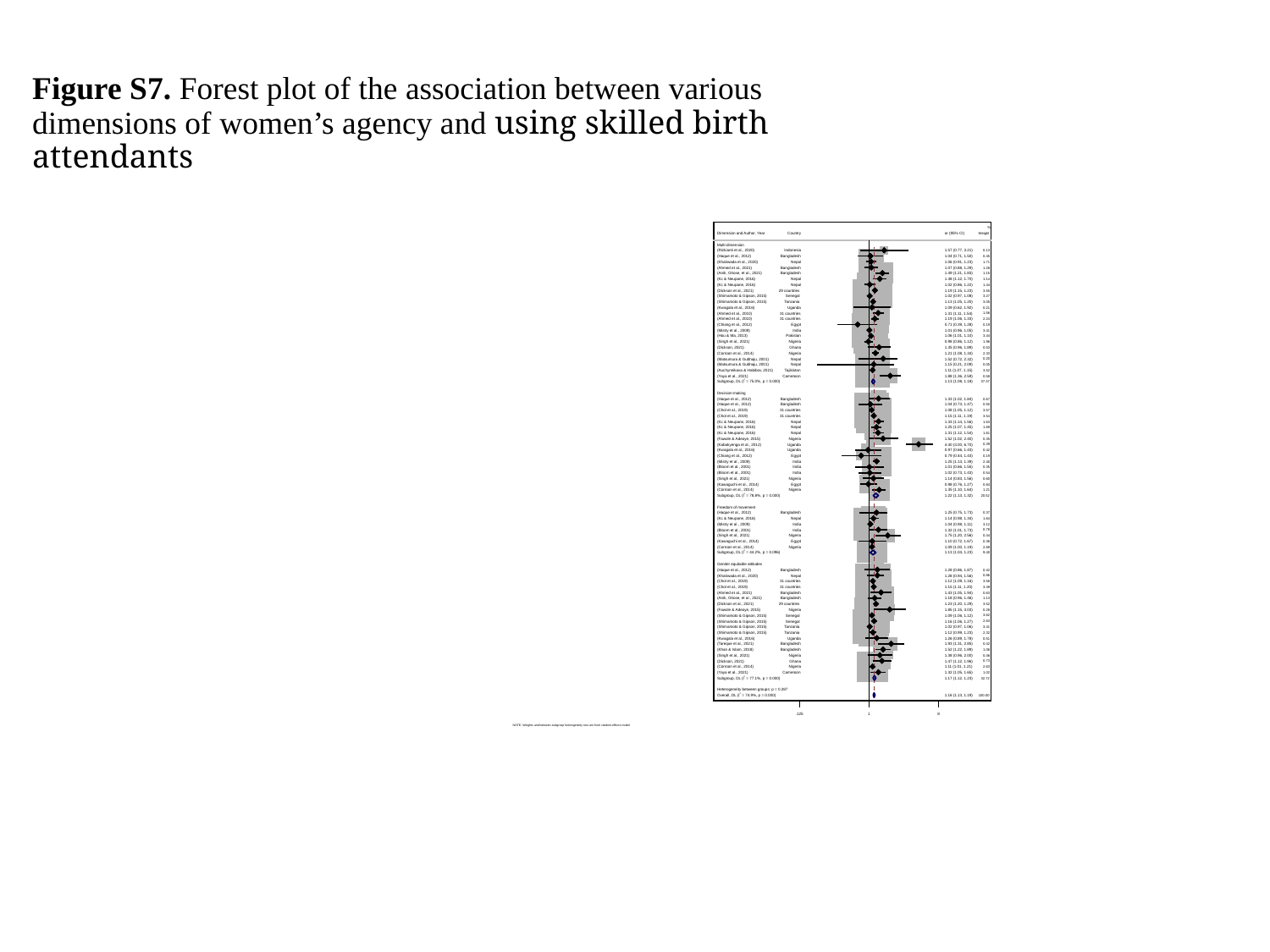

# Figure S7. Forest plot of the association between various dimensions of women’s agency and using skilled birth attendants

## Slide 9
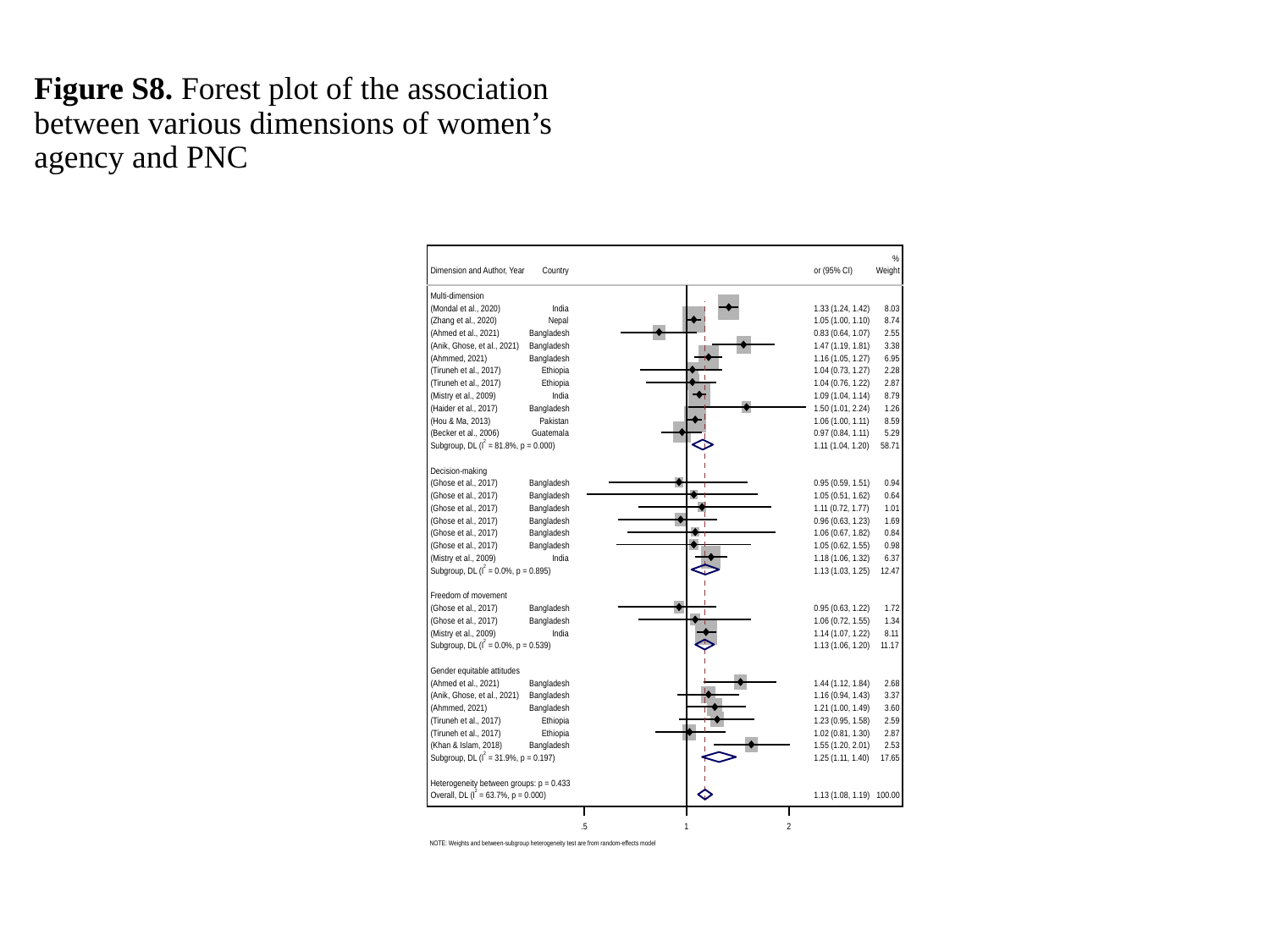

# Figure S8. Forest plot of the association between various dimensions of women’s agency and PNC
