# Supplementary material for: The Impact of Women’s Agency on Accessing and Using Maternal Healthcare Services: A Systematic Review and Meta-Analysis
Source: Int J Environ Res Public Health. 2023 Feb 23;20(5):3966. doi: 10.3390/ijerph20053966 (PMC10002172; doi:10.3390/ijerph20053966)
Supplement: Supplementary file 1 [file ijerph-20-03966-s001.zip › Supplementary file 1.pdf]

Supplementary File 1. Table S1. MMAT quality appraisal results

**Authors (year)**	4.1. Is the sampling strategy relevant to address the research question?	4.2. Is the sample representative of the target population?	4.3. Are the measurements appropriate?	4.4. Is the risk of nonresponse bias low?	4.5. Is the statistical analysis appropriate to answer the research question?
(Kareem et al., 2021)	Yes	Yes	Yes	Yes	Yes
(Rizkianti et al., 2020)	Yes	Yes	Yes	Yes	Yes
(Haque et al., 2012)	Yes	Yes	Yes	Yes	Yes
(Woldemicael, 2010)	Cannot tell	Yes	Yes	Cannot tell	Yes
(Mondal et al., 2020)	Yes	Yes	Yes	Yes	Yes
(Wado, 2018)	Yes	Yes	Yes	Yes	Yes
(Mumtaz & Salway, 2005)	Yes	Yes	Yes	Cannot tell	Yes
(Khatiwada et al., 2020)	Yes	Yes	Yes	Yes	Yes
(Obasohan et al., 2019)	Cannot tell	Yes	Yes	Cannot tell	Yes
(Zhang et al., 2020)	Yes	Yes	Yes	Cannot tell	Yes
(Tekelab et al., 2015)	Yes	Yes	Yes	Cannot tell	Yes
(Chol et al., 2019)	Yes	Yes	Yes	Cannot tell	Yes
(Ameyaw et al., 2021)	Yes	Yes	Yes	Cannot tell	Yes
(Asim et al., 2022)	Yes	Yes	Yes	Cannot tell	Yes
(Adhikari, 2016)	Cannot tell	Yes	Yes	Cannot tell	Yes
(Ghose et al., 2017)	Yes	Yes	Yes	Yes	Yes
(Furuta & Salway, 2006)	Cannot tell	Yes	Yes	Cannot tell	Yes
(Siddique et al., 2022)	Yes	Yes	Yes	Yes	Yes
(Ahmed et al., 2021)	Cannot tell	Yes	Yes	Cannot tell	Yes
(Imo, 2022)	Cannot tell	Yes	Yes	Cannot tell	Yes
(Anik, Ghose, et al., 2021)	Yes	Yes	Yes	Cannot tell	Yes
(Kc & Neupane, 2016)	Yes	Yes	Yes	Cannot tell	Yes
(Sebayang et al., 2019)	Yes	Yes	Yes	Yes	Yes
(Merrell & Blackstone, 2020)	Yes	Yes	Yes	Cannot tell	Yes
(Ntoimo et al., 2022)	Yes	Yes	Yes	Cannot tell	Yes
(Ahmmed, 2021)	Yes	Yes	Yes	Cannot tell	Yes
(Tiruneh et al., 2017)	Yes	Yes	Yes	Yes	Yes
(Dickson et al., 2021)	Yes	Yes	Yes	Cannot tell	Yes
(Shibre et al., 2021)	Yes	Yes	Yes	Cannot tell	Yes

(Gabrysch et al., 2016)	Cannot tell	Yes	Yes	Cannot tell	Yes
(Fawole & Adeoye, 2015)	Yes	Yes	Yes	Cannot tell	Yes
(Shimamoto & Gipson, 2015)	Yes	Yes	Yes	Yes	Yes
(Bello et al., 2019)	Yes	Yes	Yes	Yes	Yes
(Kabakyenga et al., 2012)	Yes	Yes	Yes	Yes	Yes
(Anderson et al., 2020)	Cannot tell	Yes	Yes	Cannot tell	Yes
(Kasymova, 2021)	Yes	Yes	Yes	Cannot tell	Yes
(Kwagala et al., 2016)	Yes	Yes	Yes	Cannot tell	Yes
(Fasina et al., 2020)	Yes	Yes	Yes	Cannot tell	Yes
(Speizer et al., 2014)	Yes	Yes	Yes	Yes	Yes
(Zaky et al., 2014)	Cannot tell	Yes	Yes	Cannot tell	Yes
(Ameyaw et al., 2016)	Yes	Yes	Yes	Cannot tell	Yes
(Tareque et al., 2021)	Yes	Yes	Yes	Yes	Yes
(Ahmed et al., 2010)	Yes	Yes	Yes	Cannot tell	Yes
(Chiang et al., 2012)	Yes	Yes	Yes	Cannot tell	Yes
(Namasivayam et al., 2012)	Yes	Yes	Yes	Yes	Yes
(Asweto et al., 2014)	Yes	Yes	Yes	Cannot tell	Yes
(Mistry et al., 2009)	Yes	Yes	Yes	Cannot tell	Yes
(Sripad et al., 2019)	Yes	Yes	Yes	Cannot tell	Yes
(Haider et al., 2017)	Yes	Yes	Yes	Cannot tell	Yes
(Sado et al., 2014)	Cannot tell	Yes	Yes	Cannot tell	Yes
(Nguyen et al., 2014)	Yes	Yes	Yes	Cannot tell	Yes
(Hou & Ma, 2013)	Yes	Yes	Yes	Cannot tell	Yes
(Ahuru, 2021)	Yes	Yes	Yes	Cannot tell	Yes
(Sohn & Jung, 2020)	Yes	Yes	Yes	Yes	Yes
(Akram et al., 2019)	Cannot tell	Yes	Yes	Cannot tell	Yes
(Bloom et al., 2001)	Yes	Yes	Yes	Yes	Yes
(Fotso et al., 2009)	Yes	Yes	Yes	Yes	Yes
(Aziz et al., 2017)	Yes	Yes	Yes	Yes	Yes
(Khan & Islam, 2018)	Yes	Yes	Yes	Yes	Yes
(Singh et al., 2021)	Yes	Yes	Yes	Cannot tell	Yes
(Yadav et al., 2021)	Cannot tell	Yes	Yes	Cannot tell	Yes

(Sserwanja et al., 2022)	Yes	Yes	Yes	Cannot tell	Yes
(Kawaguchi et al., 2014)	Yes	Yes	Yes	Yes	Yes
(Pandey et al., 2012)	Yes	Yes	Yes	Cannot tell	Yes
(Kamiya, 2011)	Yes	Yes	Yes	Cannot tell	Yes
(Dickson, 2021)	Cannot tell	Yes	Yes	Cannot tell	Yes
(Corroon et al., 2014)	Yes	Yes	Yes	Cannot tell	Yes
(Hearld et al., 2018)	Cannot tell	Yes	Yes	Cannot tell	Yes
(Gautam & Jeong, 2019)	Yes	Yes	Yes	Yes	Cannot tell
(Mokam & Zamo Akono, 2022)	Cannot tell	Yes	Yes	Cannot tell	Yes
(Matsumura & Gubhaju, 2001)	Cannot tell	Yes	Yes	Cannot tell	Yes
(Auchynnikava & Habibov, 2021)	Yes	Yes	Yes	Yes	Yes
(Story & Burgard, 2012)	Yes	Yes	Yes	Yes	Yes
(Becker et al., 2006)	Yes	Yes	Yes	Yes	Yes
(Rahman et al., 2021)	Yes	Yes	Yes	Yes	Yes
(Ali et al., 2022)	Cannot tell	Yes	Yes	Cannot tell	Yes
(Iftikhar Ul Husnain et al., 2018)	Cannot tell	Yes	Yes	Cannot tell	Yes
(Woldemicael & Tenkorang, 2010)	Yes	Yes	Yes	Cannot tell	Yes
(Yaya et al., 2021)	Yes	Yes	Yes	Cannot tell	Yes
(Bhandari et al., 2017)	Yes	Yes	Yes	Cannot tell	Yes
(Anik, Islam, et al., 2021)	Cannot tell	Yes	Yes	Cannot tell	Yes
(Shimamoto & Gipson, 2017)	Yes	Yes	Yes	Yes	Yes
